# Supplementary material for: Shared pathway-specific network mechanisms of dopamine and deep brain stimulation for the treatment of Parkinson’s disease
Source: Nat Commun. 2025 Apr 15;16:3587. doi: 10.1038/s41467-025-58825-z (PMC12000430; doi:10.1038/s41467-025-58825-z)
Supplement: Supplementary file 2 — Reporting Summary [file 41467_2025_58825_MOESM2_ESM.pdf]

nature portfolio

Corresponding author(s): Wolf-Julian Neumann

Last updated by author(s): Mar 1, 2025

Reporting Summary

Nature Portfolio wishes to improve the reproducibility of the work that we publish. This form provides structure for consistency and transparency in reporting. For further information on Nature Portfolio policies, see our Editorial Policies and the Editorial Policy Checklist.

Statistics

For all statistical analyses, confirm that the following items are present in the figure legend, table legend, main text, or Methods section.

n/a	Confirmed	
☐	☒	The exact sample size (n) for each experimental group/condition, given as a discrete number and unit of measurement
☐	☒	A statement on whether measurements were taken from distinct samples or whether the same sample was measured repeatedly
☐	☒	The statistical test(s) used AND whether they are one- or two-sided<br>*Only common tests should be described solely by name; describe more complex techniques in the Methods section.*
☐	☒	A description of all covariates tested
☐	☒	A description of any assumptions or corrections, such as tests of normality and adjustment for multiple comparisons
☐	☒	A full description of the statistical parameters including central tendency (e.g. means) or other basic estimates (e.g. regression coefficient) AND variation (e.g. standard deviation) or associated estimates of uncertainty (e.g. confidence intervals)
☐	☒	For null hypothesis testing, the test statistic (e.g. F, t, r) with confidence intervals, effect sizes, degrees of freedom and P value noted<br>*Give P values as exact values whenever suitable.*
☒	☐	For Bayesian analysis, information on the choice of priors and Markov chain Monte Carlo settings
☐	☒	For hierarchical and complex designs, identification of the appropriate level for tests and full reporting of outcomes
☐	☒	Estimates of effect sizes (e.g. Cohen's d, Pearson's r), indicating how they were calculated

Our web collection on statistics for biologists contains articles on many of the points above.

Software and code

Policy information about availability of computer code

Data collection	N/A
Data analysis	All code is made publicly available at: https://github.com/neuromodulation/manuscript-binns_cortex_stn_comm and https://zenodo.org/doi/10.5281/zenodo.10974655.<br><br>The following pieces of software were used: Python v3.11 with MNE-Python v1.6, MNE-Connectivity v0.5, MNE-BIDS v0.13, PyPARRM v1.1, PyBispectra v1.1, PTE Stats v0.2, statsmodels v0.14, NumPy v1.24, Pandas v2.0, and SciPy v1.1; MATLAB r2022b with FieldTrip r20221223, SPM12 r7771, and Lead-DBS v2.6.

For manuscripts utilizing custom algorithms or software that are central to the research but not yet described in published literature, software must be made available to editors and reviewers. We strongly encourage code deposition in a community repository (e.g. GitHub). See the Nature Portfolio guidelines for submitting code & software for further information.

Data

Policy information about availability of data

All manuscripts must include a data availability statement. This statement should provide the following information, where applicable:

- Accession codes, unique identifiers, or web links for publicly available datasets
- A description of any restrictions on data availability
- For clinical datasets or third party data, please ensure that the statement adheres to our policy

Data can be made available conditionally to data sharing agreements in accordance with data privacy statements signed by the patients within the legal framework of the General Data Protection Regulation of the European Union. Requests should be directed to the lead contact, Wolf-Julian Neumann (julian.neumann@charite.de), or the Open Data officer (opendata-neuromodulation@charite.de). Responses to such requests will be made within 1 week. The data may only be used for non-commercial purposes, and only to those persons with a valid ethics proposal for use of the data and an approved Data Privacy Impact Assessment at the Charité – Universitätsmedizin Berlin (2-7 weeks processing time). The data will be made available for 48 months, after which re-application for access is possible.

Research involving human participants, their data, or biological material

Policy information about studies with human participants or human data. See also policy information about sex, gender (identity/presentation), and sexual orientation and race, ethnicity and racism.

Reporting on sex and gender	Information about gender is reported in Table S1.<br>Patients self-reported their gender. Our results comply not only with one gender. For the cohort, both male and female patients are present. Due to the overall small sample size, sex- and gender-based analyses were not performed.
Reporting on race, ethnicity, or other socially relevant groupings	Due to the small sample size of invasive recordings, race, ethnicity and other social groupings are not reported in the current analysis.
Population characteristics	Details of the respective population characteristics are reported in Table S1.
Recruitment	All patients were undergoing invasive neurosurgery treatment and provided informed consent to participate in the respective research. All research was performed on a fully-revocable volunteer basis, completely separate from their care, with no added risk to them in any way in the event that they participated (or did not participate in research). If they chose not to participate, there was no effect on their care whatsoever. Patients were free to stop behavioural tasks at any point.
Ethics oversight	The research and brain signal recordings presented in this manuscript were performed according to the standards set by the declaration of Helsinki and after approval by the ethics committee at Charité Universitätsmedizin Berlin (EA2/129/17). All patients provided informed consent to participate in the research. The data was collected, stored, and processed in compliance with the General Data Protection Regulation of the European Union.

Note that full information on the approval of the study protocol must also be provided in the manuscript.

Field-specific reporting

Please select the one below that is the best fit for your research. If you are not sure, read the appropriate sections before making your selection.

[x] Life sciences   [ ] Behavioural & social sciences   [ ] Ecological, evolutionary & environmental sciences

For a reference copy of the document with all sections, see nature.com/documents/nr-reporting-summary-flat.pdf

Life sciences study design

All studies must disclose on these points even when the disclosure is negative.

Sample size	Sample size was chosen based on clinical inclusion criteria. The sample size is comparable to that of previous investigations.
Data exclusions	No data was excluded.
Replication	Due the novel nature of the invasive recordings, it was not possible to test the reproducibility of the findings in a different cohort.
Randomization	Due to the nature of the invasive data analysis, randomisation was not relevant for the current study.
Blinding	The investigators were not blinded to group allocation. Due to the nature of the invasive data analysis, blinding was not relevant for this study.

Reporting for specific materials, systems and methods

We require information from authors about some types of materials, experimental systems and methods used in many studies. Here, indicate whether each material, system or method listed is relevant to your study. If you are not sure if a list item applies to your research, read the appropriate section before selecting a response.

Materials & experimental systems

n/a	Involved in the study
☒	☐ Antibodies
☒	☐ Eukaryotic cell lines
☒	☐ Palaeontology and archaeology
☒	☐ Animals and other organisms
☐	☒ Clinical data
☒	☐ Dual use research of concern
☒	☐ Plants

Methods

n/a	Involved in the study
☒	☐ ChIP-seq
☒	☐ Flow cytometry
☐	☒ MRI-based neuroimaging

Clinical data

Policy information about clinical studies

All manuscripts should comply with the ICMJEguidelines for publication of clinical research and a completedCONSORT checklist must be included with all submissions.

Clinical trial registration	N/A
Study protocol	DBS implantation followed a two-step approach. In a first surgery, DBS leads were placed stereotactically after co-registering preoperative MRI and CT images. A single ECoG electrode strip was placed subdurally onto one hemisphere after minimal enlargement (~2 mm) of the frontal burr hole. The ECoG strip was aimed posteriorly toward the hand knob region of the motor cortex. The ECoG strip was placed ipsilaterally to the implantable pulse generator. All electrodes were then externalised through the burr holes via dedicated externalisation cables. Patients remained on the ward for a duration of 4-7 days until the second surgery. Between surgeries, electrophysiological recordings were performed. In the second intervention, externalisation cables of the DBS leads were replaced with permanent cables that were tunnelled subcutaneously and connected to a subclavicular implantable pulse generator. ECoG electrodes were removed via the burr hole during the second surgery.<br><br>Study participants were asked to rest comfortably in an armchair and asked not to speak or move for the duration of the recording. These rest sessions were done either under the patient's current clinical intake of dopaminergic medication (ON levodopa) or after at least 12 hours of withdrawal of all dopaminergic medication (OFF therapy). All subjects took part in the rest sessions OFF therapy and ON levodopa, and a subset of subjects took part in rest sessions after withdrawal of medication but during application of high-frequency DBS to the STN (ON STN-DBS). Contacts and stimulation parameters used during recording were determined in a monopolar clinical review. Clinically effective contacts were chosen while avoiding stimulation-induced side-effects. DBS was applied at 130 Hz with 60 µs pulse width.
Data collection	Data was collected in periods between 2020 and 2024.
Outcomes	N/A

Magnetic resonance imaging

Experimental design

Design type	Anatomical images, normative functional resting-state connectomes, and normative structural connectomes.
Design specifications	The study performed normative connectome analyses based on seeds from anatomical images normalised to MNI space.
Behavioral performance measures	No behavioural performance measures were used.

Acquisition

Imaging type(s)	Anatomical T1w/T2 and CT for electrode localisation. fMRI and dMRI from normative connectomes.
Field strength	3T
Sequence & imaging parameters	Sequence parameters for respective connectomes can be found in the original publications: fMRI connectome Ewert et al. (2018), DOI:10.1016/j.neuroimage.2017.05.015; dMRI connectome Petersen et al. (2019), DOI: 10.1016/j.neuron.2019.09.030.
Area of acquisition	Whole brain

Diffusion MRI ☒ Used ☐ Not used

Parameters: dMRI connectome parameters can be found in the original publication: Petersen et al. (2019), DOI: 10.1016/j.neuron.2019.09.030.

Preprocessing

Preprocessing software: Only preprocessed data were used as openly available through the Lead-DBS toolbox (https://lead-dbs.org) and the holographic atlas (Petersen et al., 2019, DOI: 10.1016/j.neuron.2019.09.030).

Normalization: All data were normalized using Advanced Normalization Tools as described for the default Lead-DBS pipeline in Neudorfer et al. 2023 https://doi.org/10.1016/j.neuroimage.2023.119862.

Normalization template: MNI 2009b ASYM NLIN

Noise and artifact removal: Only preprocessed data were used as openly available through the Lead-DBS toolbox and the holographic atlas.

Volume censoring: N/A

Statistical modeling & inference

Model type and settings: Linear mixed effects models with predicting fMRI/dMRI connectivity based on oscillatory coupling, using medication state as a fixed effect and subject ID as random effect.

Effect(s) tested: Correlation between fMRI/dMRI connectivity and oscillatory coupling

Specify type of analysis: ☐ Whole brain ☒ ROI-based ☐ Both

Anatomical location(s): Parcellations were defined according to the Automated Anatomical Labelling Atlas 3 (Rolls et al., 2020, DOI: 10.1016/j.neuroimage.2019.116189).

Statistic type for inference: N/A

(See Eklund et al. 2016)

Correction: N/A

Models & analysis

n/a	Involved in the study
☐	☒ Functional and/or effective connectivity
☐	☐ Graph analysis
☐	☐ Multivariate modeling or predictive analysis

Functional and/or effective connectivity: Correlation between fMRI/dMRI connectivity and oscillatory coupling

Graph analysis: *Report the dependent variable and connectivity measure, specifying weighted graph or binarized graph, subject- or group-level, and the global and/or node summaries used (e.g. clustering coefficient, efficiency, etc.).*

Multivariate modeling and predictive analysis: *Specify independent variables, features extraction and dimension reduction, model, training and evaluation metrics.*
